# Comparison of Sample Preparation and Detection Methods for the Quantification of Synthetic Musk Compounds (SMCs) in Carp Fish Samples

**DOI:** 10.3390/molecules29225444

**Published:** 2024-11-19

**Authors:** Jungmin Jo, Eunjin Lee, Na Rae Choi, Ji Yi Lee, Jae Won Yoo, Dong Sik Ahn, Yun Gyong Ahn

**Affiliations:** 1 Department of Environmental Science & Engineering, Ewha Womans University, Seoul 03760, Republic of Korea; jjm@ewhain.net (J.J.); leeej1678@kbsi.re.kr (E.L.); yijiyi@ewha.ac.kr (J.Y.L.); 2 Metropolitan Seoul Center, Korea Basic Science Institute, University-Industry Cooperation Building, 150 Bugahyeon-ro, Seodaemun-gu, Seoul 03759, Republic of Korea; 3 Department of Environmental Engineering, Kangwon National University, Chuncheon 24341, Republic of Korea; narae@kangwon.ac.kr; 4 Environmental Research Group, Korea Institute of Coastal Ecology, Inc., Bucheon 14449, Republic of Korea; jwyoo@coastkorea.com (J.W.Y.); ahndongsik@gmail.com (D.S.A.)

**Keywords:** synthetic musk compounds (SMCs), solid-phase extraction (SPE) cleanup, gas chromatograph-single quadrupole mass spectrometer (GC-SQ/MS), gas chromatograph-triple quadrupole mass spectrometer (GC-QqQ-MS/MS), carp fish samples

## Abstract

This study deals with the separation and detection methods for 12 synthetic musk compounds (SMCs), which are some of the emerging contaminants in fish samples, are widely present in environmental media, and can be considered serious risks due to their harmful effects. For the separation of co-extracted substances and the target SMCs in fish samples after ultrasonic extraction, four solid-phase extraction (SPE) sorbents were investigated. The recoveries of SMCs from 10 mL of eluent, as optimized by the elution profile, were within the acceptable range of 80–120% in all SPE types, and it was found that nitro musk and polycyclic musk compounds were separated more clearly in Florisil SPE than others (Aminopropyl, Alumina-N, PSA). Furthermore, the results of measuring the matrix effects by each SPE through the spiking experiments showed that Florisil SPE was superior. The comparison of a gas chromatograph-single quadrupole mass spectrometer (GC-SQ/MS) with selected ion monitoring (SIM) mode and GC-triple quadrupole mass spectrometer (GC-QqQ-MS/MS) with multiple reaction monitoring (MRM) modes regarding the detection method of SMCs showed that the method detection limits (MDLs) of SMCs were on average ten times lower when GC-QqQ-MS/MS with MRM mode was used. The differences between the two methods can provide essential information for selecting an analytical method in related research fields that require appropriate detection levels, such as risk assessment or pollution control.

## 1. Introduction

Synthetic musk compounds (SMCs) are consumer chemicals manufactured as fragrances and consumed in vast quantities worldwide [1,2]. As the global economy continues to grow, the consumption of SMCs is on the rise [3]. Accordingly, their use and release into the environment are becoming more frequent, and they are recognized as compounds that need attention [4]. These compounds are discharged into domestic sewage through human activities such as showering, bathing, and washing [5,6,7], are not completely removed from sewage and wastewater treatment facilities, and are released into the aquatic environment [8,9].

Comprehensive monitoring of SMCs has been performed in river waters worldwide since 2004, and the primary source has been reported to be effluents from wastewater treatment plants (WWTPs) [10,11,12,13]. Also, their concentrations detected in the rivers of many countries were ranged from 0 to 10,000 μg/L depending on the type of SMCs, and the main compounds were HHCB (4,6,6,7,8,8-hexamethyl-1,3,4,7-tetrahydrocyclopenta[g]isochromene), AHTN (1-(3,5,5,6,8,8-hexamethyl-6,7-dihydronaphthalen-2-yl)ethanone), and MK (1-(4-tert-butyl-2,6-dimethyl-3,5-dinitrophenyl)ethanone) [14,15,16,17]. As the bioaccumulation and adverse health reactions of SMCs became known, several compounds were banned and limited from use [18,19], but they are still detected in fish living in rivers, requiring constant monitoring and management [20,21,22,23].

The bioaccumulation potential and ecological risks resulting from the worldwide production, intensive use, and proven widespread distribution of SMCs have been increasingly studied in recent years. Like other well-known compounds, such as phthalates, polychlorinated biphenyls, and brominated flame retardants, SMCs are also evaluated as synthetic endocrine disruptors found in fragranced products and as molecules that can interfere with the proper functioning of the endocrine system, which can lead to harmful effects in living organisms [24]. In terms of health effects, they have been reported to be associated with dermatitis, photosensitivity, and DNA damage [25]. Moreover, they are lipophilic and have a high potential to accumulate in the body fat of organisms, so a recent attempt has been made to evaluate the metabolic impact of SMCs on obesity together with endocrine-disrupting compounds [26]. The toxicity and long-term ecological effects of SMC emission from direct (e.g., human activities) and indirect sources (e.g., WWTP effluent) extend to the marine, and studies evaluating the exposure levels of SMCs in marine organisms have raised the need to pay attention to the trophic transition of SM in the marine food chain [27]. Research on toxicity and hazards is continuously being conducted for each SMC component. Current toxicology or risk assessment research for marine organisms, including fish, is constantly undertaken for each SMC [28,29,30].

We have studied the distribution characteristics of 12 SMCs in the Han River estuary and coastal areas of Korea for several years to understand the marine environment and ecosystem [31,32]. Furthermore, an appropriate quantitative method for separation and sensitivity to measure SM concentrations in fish is needed to understand the impact on the aquatic ecosystem. To analyze different types of SMCs in various environmental matrices, several sample preparation methods such as liquid–liquid extraction, solid-phase extraction (SPE), and solid-phase microextraction (SPME) have been used to remove and separate interfering substances [33,34,35], and gas chromatography (GC) coupled with a detector such as a mass spectrometer (MS) or a tandem mass spectrometer (MS/MS), has been utilized for qualitative and quantitative analysis [1]. In the case of sample preparation using SPE or SPME, the amount of organic solvent used is small compared to the liquid–liquid extraction and is relatively simple. Still, there are limitations to the types of compounds that can be used depending on the solid cartridge or fiber [36]. This study aimed to compare and analyze the SPE sample cleanup and two detection methods for quantifying 12 SMCs in fish and to provide the analytical performance according to each approach to laboratories planning to monitor SMCs. The conditions for separating target SMCs and interfering substances well using four SPE sorbents after the ultrasonic extraction of fish samples were investigated for the sample preparation. The comparison of quadrupole MS, which is most commonly used in laboratories, and triple quadrupole MS/MS [37], which has recently been excellently used for quantitative analysis regarding the detection method, could help establish a test method to prepare for SMCs.

## 2. Results and Discussion

### 2.1. Comparison of Solid-Phase Extraction (SPE) Clean-Up

**Figure 1:**
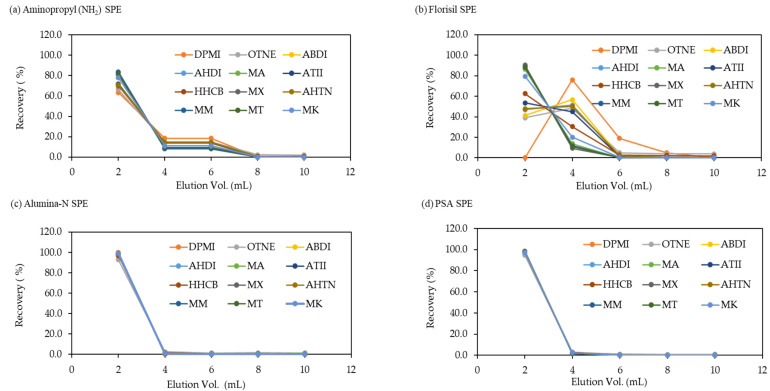
Elution patterns of 12 SMCs using four types of SPE sorbents: (**a**) Aminopropyl, (**b**) Florisil, (**c**) Alumina-N, and (**d**) Primary Secondary Amine (PSA) SPE.

The elution conditions were investigated using four SPE sorbents to separate co-extracted substances and 12 target SMCs after ultrasonic extraction of the fish sample. The elution patterns for each of the four sorbents using dichloromethane (DCM) as the elution solvent are shown in Figure 1. After loading 50 ng of SM standard mixture onto each SPE sorbent, the recoveries were investigated for every 2 mL of eluent.

For Alumina-N and PSA SPE, 12 SMCs were eluted directly into the 4 mL of solvent without being retained on the sorbents. In the case of Aminopropyl SPE, although all compounds were retained longer in the sorbent, the separation between the groups of nitro musk and polycyclic musk compounds was not clear. However, in the case of Florisil SPE, the separation between the groups of nitro musk and polycyclic musk compounds was shown more distinctly compared with the three other SPEs, while polycyclic musk compounds, especially DPMI (1,1,2,3,3-pentamethyl-2,5,6,7-tetrahydroinden-4-one) tended to be more retained in the sorbent than nitro musk compounds. From the elution pattern analytical results, it was found that 12 SMCs were completely eluted when 10 mL of DCM eluent was used in all SPE sorbents. Additionally, the recoveries for each SMC on four SPE sorbents by 10 mL of DCM eluent were investigated using 1 ng/μL of SM standard mixture.

**Figure 2:**
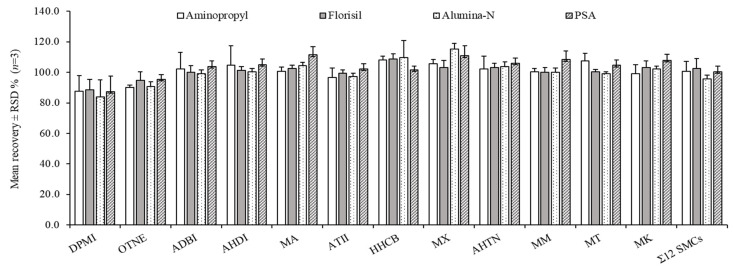
Mean recoveries and RSDs of SMCs according to the elution condition of four types of SPE sorbents (*n* = 3).

Figure 2 shows the mean recoveries of SMCs obtained by triplicate measurements according to the elution conditions of each SPE sorbent. The mean recoveries and relative standard deviations (RSDs) of Σ_12_ SMCs were 100.6 ± 6.5% for Aminopropyl SPE, 102.6 ± 6.4% for Florisil SPE, 95.6 ± 2.7% for Alumina-N SPE, and 100.4 ± 3.7% for PSA SPE, respectively. The mean recoveries ranged from 83.8% to 115.2% for all types of SPE, and RSD showed high reproducibility within 13%. Although the recovery of DPMI was relatively low at 83.8–87.5%, all SMCs met the acceptable limits of 80–120%. One of the critical factors in selecting SPE sorbents is to evaluate the matrix effects (MEs) of fish samples according to the four types of SPE cleanup. Fish samples contain lipids, proteins, amino acids, and other biomolecules, which can be extracted simultaneously during extraction [38,39,40]. Because co-extracted substances can interfere with the analysis of target chemicals, a cleanup process is essential to remove them before instrumental analysis [41,42,43].

Figure S1 compares the chromatograms obtained by the GC-SQ/MS analysis of the carp extract and 12 SMCs standard solutions. This results from overlaying the total ion chromatogram (TIC) and selected ion monitoring (SIM) chromatograms of the carp extract and 12 target SMCs to investigate the separation of target SMCs and interfering substances. The mass spectra for the major peaks of co-eluting substances with SMCs in carp extracts were shown in Figure S1a,b (lower panel), and they were identified through the NIST 2.0 library search. The main interfering substances were fatty acids, which were identified as tetradecanoic acid with retention times on GC between 8.5 and 9.3 min, pentadecanoic acid between 9.5 and 10 min, and n-hexadecanoic acid between 11 and 12 min, respectively. It can be shown that the significantly high contents of fatty acids are extracted compared to the target analytes existing at the sub-nanogram level, and in particular, the substance most likely to be affected by n-hexadecanoic acid was MK. In particular, the interferences eluted between 11 and 12 min in GC were minimized. PSA sorbent is mainly used to remove sugars, fatty acids, organic acids, lipids, and specific pigments [23,44]. Additionally, Aminopropyl, Florisil, and Alumina-N sorbents are excellent for removing lipids and effectively minimizing interfering substances in the matrix [45,46,47]. Although the results of all SPE cleanup showed the effect of removing interference substances, the Florisil sorbent exhibited lower abundances of interference peaks compared to the others. To evaluate the influence of matrix substances in the detector’s response according to four SPE sorbents, MEs were determined based on the analyte response in the presence of matrix and the analyte response in the absence of matrix. MEs are known to be caused by the loss (negative value) or enhancement (positive value) of signals for target analytes due to the competition with interferences in the active sites of the GC liner, column, and detector [48,49].

**Figure 3:**
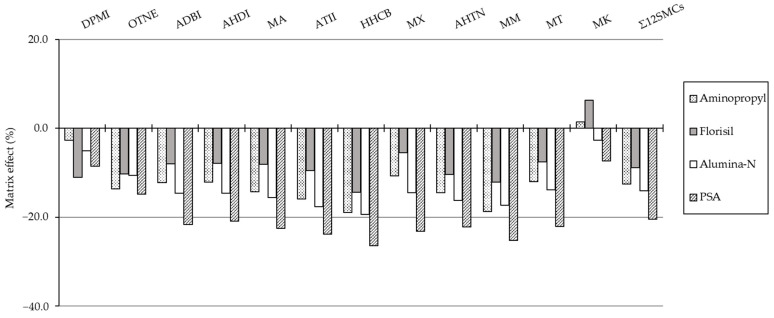
Matrix effect (%) of individual SMCs according to four SPE sorbents. The spiked concentration of the standard solution was 1 ng/μL.

ME exhibited negative values for most of the SMCs obtained by four types of SPE sorbents, except MK, corresponding to PSA and Florisil sorbents, as shown in Figure 3. The differences in ME among the four types of SPE were examined using ANOVA, and a statistically significant difference was found with *p* < 0.05. Following the ANOVA test, pairwise comparisons were conducted using the Tukey post-hoc test to determine whether there were statistically significant differences among the types of SPE sorbents. The ME results obtained with the PSA sorbent were statistically different from those obtained with the three other types of SPE sorbents. When ME (%) values are 0%, there is no ME, and the values between 20% and this value are considered to be within the acceptable range [50]. For PSA sorbent, MEs ranged from −21% to −27% for nine SMCs, excluding DPMI, OTNE, and MK. The remaining three types of SPEs showed less than 20 % (−19% to 6.4%) weak MEs for all SMCs. To summarize the results, ME (%) of Σ_12_ SMCs was low in the following order: PSA SPE (−21%) > Alumina-N SPE (−14%) > Aminopropyl SPE (−13%) > Florisil SPE (−8.8%). Florisil SPE was found to be the best cleanup when considering the reduction of interfering substances and the appropriate recovery and ME value in this study.

### 2.2. Comparison of Detection Methods

A GC-SQ/MS (single quad mass spectrometer) is the most commonly found lab instrument for targeted and untargeted analysis because it is easy to handle, robust, and cost-effective. Additionally, the SIM mode has been commonly used as a detection method for targeted analysis to quantify SMCs in GC-SQ/MS [51,52,53]. Recently, the multiple reaction monitoring (MRM) mode detection method, which provides improved sensitivity and specificity of GC-QqQ-MS/MS, has become a preferred choice as an advanced and precise quantitative detection method in various fields [52,54,55]. SMCs, which are causing concern as emerging contaminants, exist in a wide range of concentrations in environmental media, and more studies are expanding on their chemical type, geographical distribution characteristics, and toxic effects over the long term [56,57,58]. Therefore, from a monitoring perspective, comparing the two detection methods can help in selecting a quantitative analytical method.

**Figure 4:**
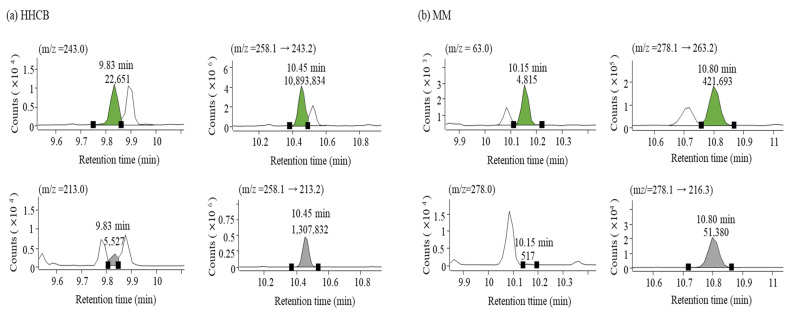
Chromatographic comparison of HHCB (**a**) and MM (**b**) obtained by GC-SQ/MS with SIM mode (left panel) and GC-QqQ-MS/MS with MRM mode (right panel) in the spiked fish samples at the concentration of 10 ng/g for HHCB and 5 ng/g for MM. The peaks of green color and gray color indicate quantifier ions and qualifier ions, respectively. Below the retention time in GC, the intensity of each peak is represented.

Table 1 shows the quantitative evaluation of 12 SMCs in the pooled blank samples using two detection methods: GC-SQ/MS with SIM mode and GC-QqQ-MS/MS with MRM mode. Linearities, limits of detection and quantitation (LOD and LOQ) for each instrument detection limit and method detection limit (MDL), accuracies, and precisions were compared. To confirm the linearity of the calibration curve, the relative response factor (RRF) and the coefficient of determination (R^2^) values were compared. RRF was used to evaluate linearity by calculating RSD % from the RRF values at each concentration obtained from the calibration curve. The RSD % for RRF of the calibration curves prepared using the two instrumental analysis methods demonstrated good linearity, remaining within 10%. The R^2^ value was high in both methods, at 0.997 or above. The linear range was also different since there was a variance in ILOD depending on each detection method. ILOD in GC-SQ/MS with SIM mode for SMCs ranged from 0.0791 ng/g to 0.151 ng/g and from 0.00935 ng/g to 0.166 ng/g in GC-QqQ-MS/MS with MRM mode, respectively. The linear range of SMCs was 10–500 ng/mL in GC-SQ/MS analysis, and the minimum and maximum concentrations in GC-QqQ-MS/MS analysis were 1–100 ng/mL, which were 10 and 5 times lower than those of GC-SQ/MS analysis. ILOD and ILOQ, representing instrumental detection limits, differed for each analyte. However, concentrations up to 15.9 times lower (in the case of HHCB) could be detected in GC-QqQ-MS/MS analysis than in GC-SQ/MS analysis. For MDL and method quantification limit (MQL) obtained by matrix spike analysis, GC-SQ/MS measurement results for each analyte were 1.03 to 4.61 ng/g and 3.10 to 18.1 ng/g, respectively. For GC-QqQ-MS/MS measurements, MDL and MQL could be measured at concentrations 10 times lower on average and up to 33 times (in the case of MK) lower than GC-SQ/MS measurement. In general, detection by QqQ-MS/MS is known as a technology that can lower the detection limit by reducing the baseline and improving the signal-to-noise ratio through double mass filtering [59]. This study compared the numerical values between the two detection methods for each SMC. QqQ-MS/MS enhances the selectivity over SQ/MS by more effectively eliminating interferences, and the results of the chromatographic comparison are shown in Figure 4.

To investigate the impact of the ME within the linear range, the slopes of the matrix and solvent, along with their ratio, were calculated and presented in Table 1. The slope ratios of the ME and solvent were 0.837–1.079 for GC-SQ/MS and 0.846–1.140 for GC-QqQ-MS/MS. Since these values fall within the acceptable range for MEs (±20%), the MEs can be disregarded [60].

Figure 4 compares the results of measuring the pooled blank samples spiked with HHCB (10 ng/g) and MM (1,1,3,3,5-pentamethyl-4,6-dinitro-2H-indene, 5 ng/g) obtained by two detection methods. HHCB and MM show differences in sensitivity related to peak area and selectivity associated with the separation from nearby interferences depending on the detection method used at the same concentration. The left panel for each analyte is the SIM chromatogram, and the right panel is the MRM chromatogram, containing each peak of quantitative (green peaks of the upper panel) and qualitative (gray peaks of the lower panel) ions. In both HHCB and MM, the peak area of the quantitative ion in MRM mode was tens to hundreds of times higher than that in SIM mode. In the case of the qualitative ion, the separation from the nearby peaks was clear, allowing for accurate identification.

The MDLs of the proposed analytical method are presented in Table S1, compared with those reported in other studies. Although the MDL varies depending on fish species, fat content, and sample size, the MDLs were comparable to or lower than those obtained using Pressurized Liquid Extraction (PLE) and QuEChERS methods [23,61,62].

Apparent recovery (R_app_ %) and precision were measured in triplicate by spiking the corresponding concentration four times higher than the ILOQ of each SMC into the pooled blank samples, and the results are shown in Table 2.

The apparent recoveries of all SMCs were 79.9–113% (RSD 5.4–22%) in GC-SQ/MS analysis and 83.0–117% (RSD 1.4–17%) in GC-QqQ-MS/MS analysis. In both detection methods, all SMCs show acceptable accuracy and precision with recoveries within 70–120% and RSD less than 25%. The mean recoveries of 12 SMCs were 97.8% (RSD 3.9%) in GC-SQ/MS analysis and 97.6% (RSD 2.6%) in GC-QqQ-MS/MS analysis. The mean difference in R_app_ (%) values in both analytical methods, as determined by a *t*-test, showed no statistically significant difference (*p* = 0.889 > 0.05). From these results, the proposed optimal sample preparation can be combined with both detection methods; however, since the detection limits are different, it suggests that the selection of the detection method should depend on the purpose of the analysis, such as the monitoring for regulation and risk assessment.

### 2.3. Application to Real Samples

Two optimized detection methods combined with Florisil cleanup were used to determine the level of SMCs in fish collected from the estuary and coastal areas near the Han River in South Korea. To compare the two methods, a positive fish sample containing various compounds and having concentrations above a certain level (>MQL) of SMCs was selected and collected between Nanji and Seonam wastewater treatment plants. Figure S2a–c illustrate the comparison results between the two methods for SMCs detected over various concentrations in a positive sample.

When the quantifying concentration was categorized into low (−20 ng/g), medium (75–120 ng/g), and high (2000–3000 ng/g) levels, there were compounds detected only in GC-QqQ-MS/MS analysis, such as AHDI 1-(6-tert-butyl-1,1-dimethyl-2,3-dihydroinden-4-yl)ethenone), at low concentrations, and the agreement between the two methods for five compounds was evaluated excluding those compounds (Figure S2d). The agreement between the two methods for the five compounds ranged from 80.3% to 116%, as shown in Figure S2d, indicating that the methods were interchangeable. The agreement (%) shown in Figure S2d was calculated based on the values measured by GC-QqQ-MS/MS analysis. The difference between SMC concentrations measured from the two detection methods was analyzed using a nonparametric Wilcoxon Signed-Rank test. This approach is commonly used to statistically compare two matching results and is considered significant when the *p*-value is less than 0.05 [63]. The two paired data of SMCs detected in the sample obtained by the two methods were compared, and the *p*-value was greater than 0.05, indicating that there was no significant difference.

Due to the difference in MDL between the two methods, AHDI was not detected by GC-SQ/MS analysis but was quantified by GC-QqQ-MS/MS analysis. As shown in Figure S3, the chromatogram of AHDI showed a low signal below the MDL in GC-SQ/MS with SIM mode, while it was quantified at a concentration of 0.357 ng/g in GC-QqQ-MS/MS with MRM mode.

## 3. Materials and Methods

### 3.1. Reagents and Standards

The SMCs analyzed in this study were six types of nitro musk compounds and six types of polycyclic musk compounds, and their information (name, abbreviation, CAS No., molecular weight, molecular formula, log K_ow_, and chemical structure) were as shown in Table S2. The neat standards of nitro musk compounds and AHTN were purchased from Sigma Aldrich (St. Louis, MO, USA). MX (1-tert-butyl-3,5-dimethyl-2,4,6-trinitrobenzene) and MK were the standard solutions dissolved in acetonitrile (ACN) at 100 μg/mL concentration. Polycyclic musk compounds, except HHCB and AHTN, were purchased from Toronto Research Chemicals (Toronto, ON, Canada). HHCB was purchased from HPC Standards GmbH (Cunnersdorf, Germany) and supplied in cyclohexane at 100 μg/mL concentration. Fluoranthene-d_10_ (Fla-d_10_) was used as an internal standard purchased from Sigma-Aldrich (St. Louis, MO, USA). Anhydrous sodium sulfate (Na_2_SO_4_) from Fuji-film Wako Pure Chemical Corporation (Osaka, Japan) was used. The ACN, DCM, and n-hexane (n-Hex) were of HPLC grade (Fisher Scientific, Loughborough, UK).

### 3.2. Sample Pretreatment

**Figure 5:**
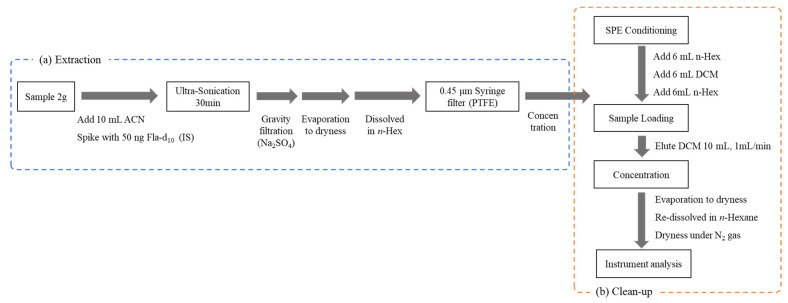
Flow chart of the analytical procedure: (**a**) Extraction and (**b**) Cleanup.

The crucian carp is a representative fish species widely distributed and consumed in Korea. It has been commonly used as an indicator to evaluate the bioaccumulation impact on aquatic organisms of pollutants with bioaccumulation potential, such as cyclic and linear siloxanes [64], hexabromocyclododecanes (HBCDs) [65], phenolic compounds, polychlorinated naphthalenes (PCNs) [66], and polychlorinated biphenyls (PCBs) [67]. The edible parts of the crucian carps, which did not contain the 12 SMCs, were homogenized to prepare a 500 g pooled sample (pooled blank sample) collected from a clean environment and used in the spiking experiment for the development, optimization, and validation of the analytical method. The fat content of the crucian carp was 1.20 ± 0.27% in triplicate analysis. A 50 ng of Fla-d_10_ was spiked into a 2 g homogenized sample, and ultrasonic extraction was performed with 10 mL of ACN. ACN was selected as the extraction solvent to minimize matrix interference, as it effectively reduces the extraction of lipophilic substances, waxes, fats, and pigments [68,69]. Compared with 20, 30, 40, and 60 min for the extraction, the optimum extraction time was 30 min when considering the best extraction efficiency and other interferences. After extraction, the solvent containing the target SMCs was filtered through 5 g of anhydrous Na_2_SO_4_ using qualitative filter paper (No. 2, ADVANTEC Toyo Kaisha, Ltd., Tokyo, Japan). The ultrasonic extraction and filtration process was repeated three times to collect 30 mL of ACN. The collected solvent was evaporated using a rotary evaporator (Eyela, Tokyo, Japan) until the ACN was completely dry. Four SPE sorbents for cleanup were evaluated: Aminopropyl, Florisil, Alumina-N, and PSA. The main characteristics of the four SPE sorbents are summarized in Table S3. First, the PSA was conditioned with 6 mL of MeOH, followed by 6 mL of DCM. Other types of three SPE were conditioned with 6 mL of n-Hex and 6 mL of DCM. The concentrated extract was loaded onto the upper surface of the conditioned SPE sorbents and eluted with 10 mL of DCM at a rate of 1 mL/min. Finally, the elution was evaporated and then reconstituted with 50 μL before GC injection. The entire sample preparation is illustrated in the flow chart (Figure 5).

### 3.3. Instrumental Analysis

The performance of two instrument systems, GC-SQ/MS with SIM mode and GC-QqQ-MS/MS with MRM mode, was compared for the quantification of 12 SMCs in fish samples. A GC-SQ/MS was performed by an Agilent 7890B gas chromatograph equipped with a 5977A mass selective detector spectrometer system (Agilent, Palo Alto, CA, USA). The column used was a DB-5MS UI capillary column (5% diphenyl, 95% dimethylsiloxane phase, 30 m × 0.25 mm × 0.25 μm) from J&W Scientific (Folsom, CA, USA). The sample was injected at 1 μL in splitless mode at 280 °C. Helium as carrier gas (99.999%) flow was 1 mL/min. The following GC-SQ/MS oven temperature program was applied: the initial temperature was set to 60 °C, ramped up at 20 °C/min to 200 °C, then increased at 20 °C/min to 220 °C, and finally ramped up at 20 °C/min to 320 °C, where it was held for 10 min. A GC-QqQ-MS/MS used a model that combines Agilent’s 7890B GC and 7010 Triple Quad MSD. The analytes were separated using the same column and oven conditions as in GC-SQ/MS. High-purity helium was used as the carrier gas, and nitrogen was used as the collision gas, with flow rates of 1 mL/min and 1.5 mL/min, respectively. The analytes were measured using SIM mode for qualitative and quantitative ions in GC-SQ/MS analysis. The selection of quantifier and qualifier ions was based on the fragmented ions of the first and second most abundance ions, respectively, for GC-QqQ-MS/MS with MRM mode; optimal precursor ion and product ions were selected by adjusting the appropriate collision energy (CE) values. Product ions for quantification and qualification were selected based on their abundance in a composite product ion scan spectrum obtained for the precursor ion of each analyte at multiple CEs. The optimized acquisition parameters for each of the 12 SMCs in GC-SQ/MS with SIM mode and GC-QqQ-MS/MS with MRM mode are shown in Table 3.

The IS and 12 SMCs measured under the instrumental analysis conditions described above were detected within the retention time ranges of 7.04–13.01 min (GC-SQ/MS) and 7.37–13.92 min (GC-QqQ-MS/MS) (Figure S4).

### 3.4. Quality Assurance and Quality Control (QA/QC)

All analytical results conducted to assess method validation were equally applied to GC-SQ/MS and GC-MS/MS-QqQ equipment and then compared.

The linearity of the calibration curve was verified by checking the coefficient of determination (R^2^) and the RSD value of RRF. The acceptable range for the RSD value of RRF was set to be within ±20%. The detection and quantification limits of the instrument (ILOD and ILOQ) were calculated by measuring the standard substance at the lowest concentration level on the calibration curve 6 times and multiplying the standard deviation of the measured values by 3 and 10, respectively. To calculate the MDL, a low concentration of the standard substance, approximately 3 to 5 times the ILOD, was spiked into fish samples and analyzed in seven replicates. The MDL was determined by multiplying the standard deviation of the seven spiked concentrations by 3.14 (the *t*-test value for 6 degrees of freedom) [70]. The MQL was calculated by multiplying the MDL value by 3 [71]. The recoveries (Figure 2) were not done using the samples according to the elution condition of SPE sorbents. They were assessed by comparing the theoretical concentration with the solution eluted with 10 mL of DCM after loading the standard into each SPE sorbent without the internal standard. This was done to ensure all analytes were eluted with 10 mL of DCM. To measure apparent recovery, 2 g of pooled blank samples spiked with the known amount of the analytes were prepared (Table 2) and determined by the analytical procedure, as shown in Figure 5.

The ME was assessed using the following equation [72]:

ME (%) = (C_Post-Extr_/C_STD_ − 1) × 100

C_Post-Extr_ represents the analyte concentration obtained from the spiked extract minus the analyte concentration in the non-spiked extract after ultrasonication and SPE without IS spiking. C_STD_ is the standard solution of the same concentration.

### 3.5. Statistical Analysis

Statistical analysis was performed using the SPSS version 25.0 software program (IBM, Inc. Chicago, IL, USA). One-way analysis of variance (ANOVA), *t*-test, and Wilcoxon rank-sum test were used to evaluate the significance of the measured differences. After conducting the ANOVA analysis, a Tukey post-hoc test was performed. The statistical data employed the ME (%), R_app_ (%), and the measured results obtained from the two analytical methods. All statistical analyses were two-sided, with significance at *p* < 0.05. A *p*-value lower than 0.05 was accepted as statistically significant.

## 4. Conclusions

For the quantification of SMCs in fish samples, four SPE sorbents were examined as cleanup methods, and GC-SQ/MS with SIM mode and GC-QqQ-MS/MS with MRM mode were investigated as detection methods. When the appropriate elution volume was set to 10 mL of each sorbent, and the elution profiles were compared, it was found that SPE cleanup that could clearly separate nitro musk and polycyclic musk compounds was Florisil sorbent. The recovery results of each SPE satisfied the acceptable range of 80–120% for all types of SPE, the ME results of PSA sorbent showed statistically significant differences from other SPEs. However, Florisil SPE, which had the lowest ME value (%) of SPE sorbents among the three groups, was selected as a cleanup method.

The information in the analysis of data related to detection limits (ILOD, ILOQ, MDL, MQL) of two mass spectrometric methods for quantifying SMCs helps determine which analytical method to apply in research fields (risk assessment or pollution control, etc.). The linear ranges were determined using two methods, and although they differed depending on the compounds, MDL and MQL were measured to be up to 33 times lower when GC-QqQ-MS/MS was used than in GC-SQ/MS analysis. The limitations of GC-SQ/MS analysis, which is commonly used, were confirmed through the analysis of real samples, and the adequate range for monitoring was presented in this study. However, the MDL obtained by the proposed GC-QqQ-MS/MS method was superior to the results of previous studies.

## Figures and Tables

**Table 1:** Linearities, slopes of solvent and matrix, instrumental limits of detection and quantification, and method limits of detection and quantification of 12 analytes in fish samples analyzed using (**a**) GC-SQ/MS and (**b**) GC-QqQ-MS/MS.

**(a) ** **GC-SQ/MS**										
**Analyte**	**Linearity**			**Solvent ** **Slope**	**Matrix ** **Slope**	**Matrix Slope** **/Solvent Slope**	**ILOD ** **(ng/g)**	**ILOQ ** **(ng/g)**	**MDL ** **(ng/g)**	**MQL** **(ng/g)**
	**Range (ng/mL)**	RRF * RSD (%)	**R^2^**							
DPMI	10–500	6.1	0.998	0.405	0.339	0.837	0.151	0.504	1.46	4.37
OTNE	10–500	10	0.998	0.161	0.144	0.895	0.0955	0.318	1.23	3.68
ADBI	10–500	9.5	0.998	0.282	0.258	0.914	0.0869	0.290	1.20	3.59
AHDI	10–500	11	0.998	0.435	0.406	0.933	0.0828	0.276	1.34	4.02
MA	10–500	6.8	0.999	0.181	0.188	1.037	0.265	0.884	2.18	6.55
ATII	10–500	7.0	0.999	0.370	0.356	0.962	0.0979	0.326	1.67	5.01
HHCB	10–500	8.8	1.000	0.105	0.113	1.079	0.228	0.759	3.44	10.3
MX	10–500	5.8	0.998	0.213	0.207	0.970	0.190	0.634	3.29	9.87
AHTN	10–500	6.8	0.999	0.124	0.121	0.981	0.109	0.364	4.61	13.8
MM	10–500	8.1	0.998	0.342	0.348	1.019	0.0791	0.264	1.33	3.98
MT	10–500	5.6	0.997	0.289	0.279	0.965	0.114	0.381	1.03	3.10
MK	10–500	4.1	0.997	0.211	0.210	0.998	0.246	0.820	6.02	18.1
**(b) GC-QqQ-MS/MS**										
**Analyte**	**Linearity**			**Solvent ** **Slope**	**Matrix** **Slope**	**Matrix Slope** **/Solvent Slope**	**ILOD ** **(ng/g)**	**ILOQ ** **(ng/g)**	**MDL** **(ng/g)**	**MQL ** **(ng/g)**
	**Range (ng/mL)**	**RRF RSD (%)**	**R^2^**							
DPMI	1–100	9.0	1.000	2.536	2.144	0.846	0.0116	0.0388	0.087	0.262
OTNE	2–100	8.8	0.997	0.701	0.720	1.027	0.0282	0.0940	0.552	1.66
ADBI	1–100	19	0.999	0.422	0.402	0.953	0.0126	0.0421	0.161	0.483
AHDI	1–100	12	0.998	0.411	0.450	1.094	0.0127	0.0424	0.175	0.525
MA	5–100	8.0	1.000	0.200	0.226	1.126	0.0188	0.0627	0.280	0.841
ATII	5–100	2.0	1.000	0.313	0.357	1.140	0.0315	0.105	1.02	3.05
HHCB	1–100	17	0.999	3.092	3.452	1.117	0.0143	0.0478	0.665	1.99
MX	8–100	11	1.000	0.088	0.094	1.066	0.166	0.553	0.944	2.83
AHTN	1–100	11	0.999	5.703	6.496	1.139	0.00935	0.0312	0.171	0.512
MM	5–100	13	0.999	0.278	0.315	1.133	0.046	0.153	0.350	1.05
MT	5–100	9.7	1.000	1.095	1.208	1.103	0.0150	0.0499	0.193	0.579
MK	5–100	14	0.999	0.025	0.023	0.918	0.106	0.354	0.183	0.550

* RRF: Relative response factor.

**Table 2:** Accuracy (R_app_ %) and precision (RSD %) of 12 SMCs in spiked pooled blank samples obtained by GC-SQ/MS and GC-QqQ-MS/MS.

Analyte	GC-SQ/MS (*n* = 3)			GC-QqQ-MS/MS (*n* = 3)		
	Spking Level (ng)	R_app_ (%)	RSD (%)	Spking Level (ng)	R_app_ (%)	RSD (%)
DPMI	4.0	113	14	0.40	83.0	3.4
OTNE	4.0	114	4.8	2.0	108	9.3
ADBI	4.0	82.1	1.9	0.40	102	17
AHDI	4.0	97.0	5.4	0.40	96.1	7.7
MA	10	105	10	1.0	100	5.5
ATII	4.0	103	3.3	4.0	91.4	17
HHCB	10	88.9	22	0.40	81.9	12
MX	10	94.9	13	4.0	117	10
AHTN	10	79.9	16	0.20	115	13
MM	4.0	94.1	17	4.0	102	9.5
MT	4.0	94.1	9.0	1.0	89.3	15
MK	10	112	8.0	4.0	84.2	1.4
$\Sigma_{12}$ SMCs		97.8	3.9		97.6	2.6

**Table 3:** Optimized acquisition parameters of GC-SQ/MS with SIM mode and GC-QqQ-MS/MS with MRM mode for 12 target SMCs analysis.

Analyte	GC-SQ/MS with SIM Mode		GC-QqQ-MS/MS with MRM Mode		
	Qualifier Ion (*m*/*z*)	Quantifier Ion (*m*/*z*)	Precursor Ion (*m*/*z*)	Qualifier Ion (*m*/*z*) [CE * (V)]	Quantifier Ion (*m*/*z*) [CE (V)]
DPMI	206.0	191.0	206.1	191.1 (5)	163.1 (5)
OTNE	119.0	191.0	191.1	109.2 (15)	121.0 (15)
ADBI	244.0	229.0	244.0	173.1 (15)	229.2 (15)
AHDI	244.0	229.0	244.1	187.1 (15)	229.2 (15)
MA	268.0	253.0	268.1	91.0 (50)	253.1 (5)
ATII	258.0	215.0	258.2	131.1 (30)	173.1 (15)
HHCB	213.0	243.0	258.1	213.2 (15)	243.2 (5)
MX	128.0	282.0	297.2	77.1 (50)	282.1 (5)
AHTN	201.0	159.0	258.1	187.1 (15)	243.2 (5)
MM	278.0	263.0	278.1	216.3 (15)	263.2 (5)
MT	266.0	251.0	266.1	91.1 (50)	251.1 (5)
MK	294.0	279.0	294.3	91.0 (50)	189.1 (5)
Fla-d_10_	106.0	212.0	106.2	78.0 (15)	92.1 (15)

* CE: Collision energy.

## Data Availability

The data presented in this study are available on request from the corresponding author.

## Supplementary Materials

The following supporting information can be downloaded at https://www.mdpi.com/article/10.3390/molecules29225444/s1, Table S1: Comparison of MDLs of the proposed method with previous literature for the SMC analysis in fish samples; Table S2: Chemical names, abbreviations, CAS numbers, molecular formulas, molecular weights, log K_ow_ and chemical structures of the 12 SMCs classified into two groups; Table S3: List and the properties of the four SPE sorbents used in this study; Figure S1: TIC (a) and SIM (b) overlay chromatograms obtained by the GC-SQ/MS analysis of the carp extract (scan mode, black color) and 12 SMCs standard solutions (SIM mode, blue color). The red boxes in the lower panel indicate the three major interferences that were eluted at similar times on the GC column as the target analytes. Each interfering substance was identified by NIST 2.0 library searching of its individual mass spectrum; Figure S2: Comparison of individual SMCs quantifying results detected in a positive sample obtained by GC-SQ/MS with SIM mode and GC-QqQ-MS/MS with MRM mode. (a), (b) and (c) compare the results of detected SMCs according to the concentration range. (d) represents the percentage of agreement between two methods for the concentration results of the mainly detected SMCs; Figure S3: Chromatograms of AHDI obtained by (a) GC-SQ/MS with SIM mode (not detected < MDL) and (b) GC-QqQ-MS/MS with MRM mode at the concentration of 0.357 ng/g; Figure S4: Typical chromatograms of the standard solutions with 12 SMCs and Fla-d_10_ (IS) obtained by GC-SQ/MS with SIM mode and GC-QqQ-MS/MS with MRM mode. Refs. [73,74,75] are cited in the Supplementary Materials.
