# Supplementary material for: Comparison of Sample Preparation and Detection Methods for the Quantification of Synthetic Musk Compounds (SMCs) in Carp Fish Samples
Source: Molecules. 2024 Nov 19;29(22):5444. doi: 10.3390/molecules29225444 (PMC11597492; doi:10.3390/molecules29225444)
Supplement: Supplementary file 1 [file molecules-29-05444-s001.zip › molecules-3268282-supplementary.pdf]

- Supplementary Material –

Comparison of Sample Preparation and Detection Methods for the Quantification of Synthetic Musk Compounds (SMCs) in Carp Fish Samples

Jungmin Jo [1,†], Eunjin Lee [1,2,†], Na Rae Choi [3], Ji Yi Lee [1], Jae Won Yoo [4], Dong Sik Ahn [4] and Yun Gyong Ahn [2,*]

[1] Department of Environmental Science & Engineering, Ewha Womans University, Seoul 03760, Republic of Korea; jjm@ewhain.net (J.J.); leeej1678@kbsi.re.kr (E.L.); yijiyi@ewha.ac.kr (J.Y.L.)
[2] Metropolitan Seoul Center, Korea Basic Science Institute, University-Industry Cooperation Building, 150 Bugahyeon-ro, Seodaemun-gu, Seoul 03759, Republic of Korea
[3] Department of Environmental Engineering, Kangwon National University, Chuncheon 24341, Republic of Korea; narae@kangwon.ac.kr
[4] Environmental Research Group, Korea Institute of Coastal Ecology, Inc., Bucheon 14449, Republic of Korea; jwyoo@coastkorea.com (J.W.Y.), ahndongsik@gmail.com (D.S.A.)
* Correspondence: ygahn@kbsi.re.kr
† These authors contributed equally to this work.

Contents:

Table S1. Comparison of MDL levels of the proposed method with previously reported literature on SMC analysis in fish

Table S2. Chemical names, abbreviations, CAS numbers, molecular formulas, molecular weights, log Kow and chemical structures of the 12 SMCs classified into two groups

Table S3. List and the properties of the four SPE sorbents used in this study

Figure S1. TIC (a) and SIM (b) overlay chromatograms obtained by the GC-SQ/MS analysis of the carp extract (scan mode, black color) and 12 SMCs standard solutions (SIM mode, blue color). The red boxes in the lower panel indicate the three major interferences that were eluted at similar times on the GC column as the target analytes. Each interfering substance was identified by NIST 2.0 library searching of its individual mass spectrum

Figure S2. Comparison of individual SMCs quantifying results detected in a positive sample obtained by GC-SQ/MS with SIM mode and GC-QqQ-MS/MS with MRM mode. (a), (b) and (c) compare the results of detected SMCs according to the concentration range. (d) represents the percentage of agreement between two methods for the concentration results of the mainly detected SMCs

Figure S3. Chromatograms of AHDI obtained by (a) GC-SQ/MS with SIM mode (not detected < MDL) and (b) GC-QqQ-MS/MS with MRM mode at the concentration of 0.357 ng/g

Figure S4. Typical chromatograms of the standard solutions with 12 SMCs and Fla-d_{10} (IS)

Table S1. Comparison of MDL levels of the proposed method with previously reported literature on SMC analysis in fish

Sample	Sample weight (g)	Method	Target compound number	MDL (ng/g)	Ref.
crucian carp (Carassius auratus)	2	Ultrasonication (Extraction)<br>Florisil SPE (clean up)<br>GC-QS/MS and GC-QqQ-MS/MS	12	1.03-6.02<br>(GC-QS/MS)<br>0.087-1.02<br>(GC-QqQ MS/MS)	This study
Bluegill (muscle tissue only ~0.4% lipid)	1	Silicagel columm<br>GC–SIM–MS	5	4.0-17	[1]
Sonora sucker (muscle, skin and belly flap tissue ~4.9% lipid)	1	Silicagel columm<br>GPC (gel-permeation chromatography)<br>GC–MS/MS	5	12-397	
Red mullet (Mullus surmuletus)	0.5	PLE (Pressurised liquid extraction)<br>QuEChers<br>GC-IT-MS/MS	11	0.25-5 (PLE)<br>0.25-10<br>(QuEChers)	[2]
codfish (Gadus morhua), sole (Solea, solea) and hake (Merluccius merluccius)	0.25	GC-MS/MS - SPME Arrow	8	0.5-2.5	[3]
hake and salmon	0.5	QuEChERS<br>Florisil (dSPE)<br>GC-IT-MS/MS	10	1-5 (hake)<br>1-5 (salmon)	[4]
Tilapia, Smallmouth bass and Bream	~ 2.5	PLE (Pressurised liquid extraction)<br>Silica<br>In-cellclean-up<br>GPC (gel-permeation chromatography)<br>GC-MS/MS	5	8-360 (Tilapia)<br>1.6-38 (Bream)	[5]

Table S2. Chemical names, abbreviations, CAS numbers, molecular formulas, molecular weights, log K_{ow} and chemical structures of the 12 SMCs classified into two groups

Category	IUPAC name	Trade name	Abbreviation	CAS No.	Molecular formular	Molecular weight (g/mol)	log K_{ow}	Chemical structures
	1-tert-butyl-3,4,5-trimethyl-2,6-dinitrobenzene	Musk tibetene	MT	145-39-1	$C_{13}H_{18}N_2O_4$	266.3	5.9[a]	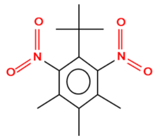
	1-tert-butyl-2-methoxy-4-methyl-3,5-dinitrobenzene	Musk ambrette	MA	83-66-9	$C_{12}H_{16}N_2O_5$	268.3	4.7[a]	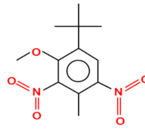
Nitro musks	1,1,3,3,5-pentamethyl-4,6-dinitro-2H-indene	Musk moskene	MM	116-66-5	$C_{14}H_{18}N_2O_4$	278.3	6.1[a]	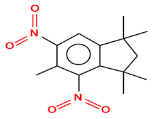
	1-(4-tert-butyl-2,6-dimethyl-3,5-dinitrophenyl)ethanone	Musk ketone	MK	81-14-1	$C_{14}H_{18}N_2O_5$	294.3	4.24[b]	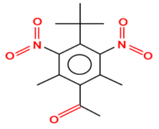
	1-tert-butyl-3,5-dimethyl-2,4,6-trinitrobenzene	Musk xylene	MX	81-15-2	$C_{12}H_{15}N_3O_6$	297.3	5[a]	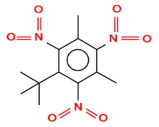

	1,1,2,3,3-pentamethyl-2,5,6,7-tetrahydroinden-4-one	Cashmeran	DPMI	33704-61-9	$C_{14}H_{22}O$	206.3	4.2[b]	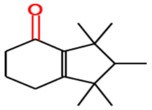
	1-(6-tert-butyl-1,1-dimethyl-2,3-dihydroinden-4-yl)ethanone	Celestolide	ADBI	13171-00-1	$C_{17}H_{24}O$	244.4	5.7[b]	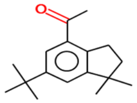
	1-(1,1,2,3,3,6-hexamethyl-2H-inden-5-yl)ethanone	Phantolide	AHDI	15323-35-0	$C_{17}H_{24}O$	244.4	5.59[be]	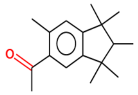
Polycyclic musks	1-(1,1,2,6-tetramethyl-3-propan-2-yl-2,3-dihydroinden-5-yl)ethanone	Traesolide	ATII	68140-48-7	$C_{18}H_{26}O$	258.4	6.3[a]	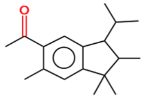
	4,6,6,7,8,8-hexamethyl-1,3,4,7-tetrahydrocyclopenta[g]isochromene	Galaxolide	HHCB	1222-05-5	$C_{18}H_{26}O$	258.4	5.3[b]	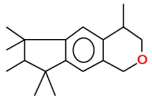
	1-(3,5,5,6,8,8-hexamethyl-6,7-dihydronaphthalen-2-yl)ethanone	Tonalid	AHTN	1506-02-1	$C_{18}H_{26}O$	258.4	5.7[b]	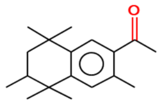
	1-(2,3,8,8-tetramethyl-1,3,4,5,6,7-hexahydronaphthalen-2-yl)ethanone	Iso E Super	OTNE	54464-57-2	$C_{16}H_{26}O$	234.4	5.65[b]	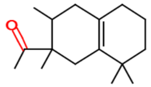

[a][6] , [b] https://echa.europa.eu/information-on-chemicals

Table S3. List and the properties of the four SPE sorbents used in this study

Sorbent phase type	Normal-Phase			Ion-Exchange
Bed sorbent	Aminopropyl (-NH_2)	Florisil	Alumina-N (neutral)	Primary Secondary Amine (PSA)
Structure of bonded phase	-$(CH_2)NH_2$	Mg_2SiO_3	Al_2O_3	ethylenediamine-N-propyl
Sorbent amount/cartridge volume	500mg / 6cc	1g / 6cc	1g / 6cc	500mg /6cc
Particle Size	55 - 105 µm	50 - 200 µm	50 - 300 µm	50 µm
Pore Size	125 Å	60 Å	120 Å	70 Å
Distributor	Waters (Milford, MA, USA)	Waters (Milford, MA, USA)	Waters (Milford, MA, USA)	Sigma-Aldrich (St. Louis, MO, USA)

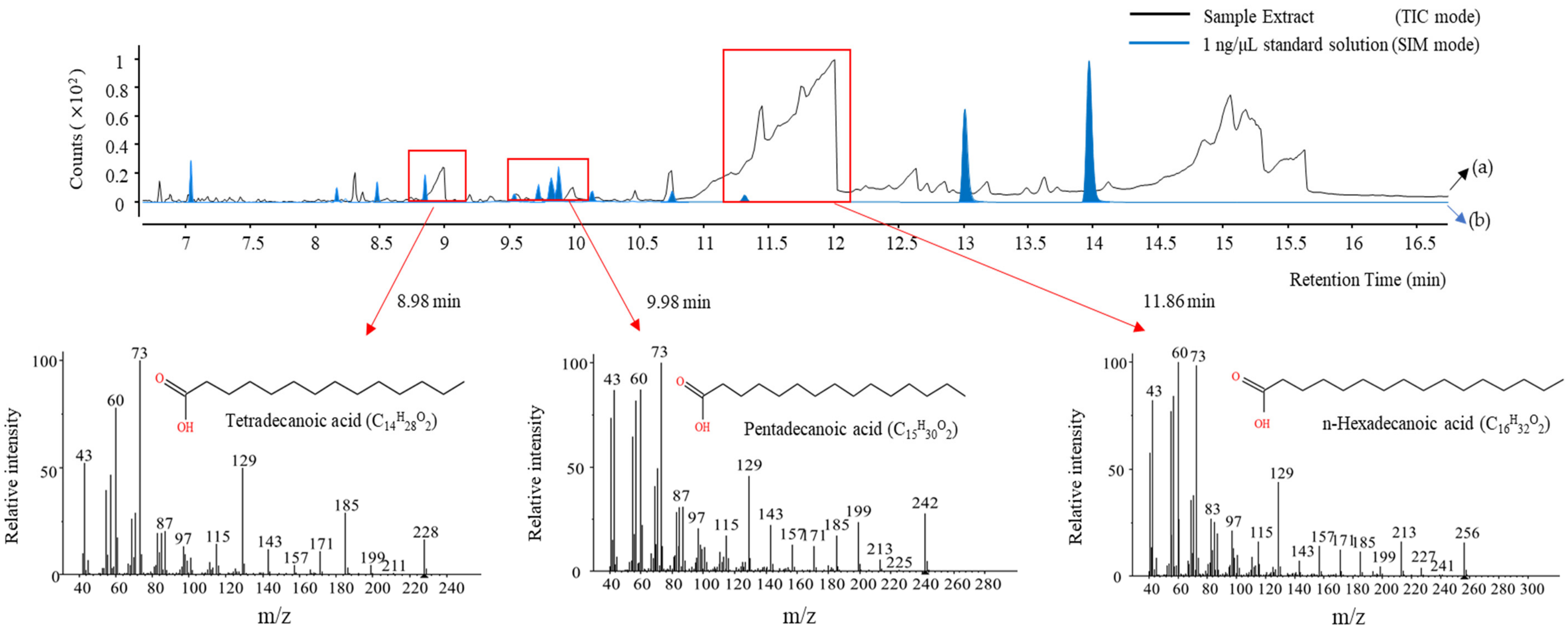

Figure S1. TIC (a) and SIM (b) overlay chromatograms obtained by the GC-SQ/MS analysis of the carp extract (scan mode, black color) and 12 SMCs standard solutions (SIM mode, blue color). The red boxes in the lower panel indicate the three major interferences that were eluted at similar times on the GC column as the target analytes. Each interfering substance was identified by NIST 2.0 library searching of its individual mass spectrum.

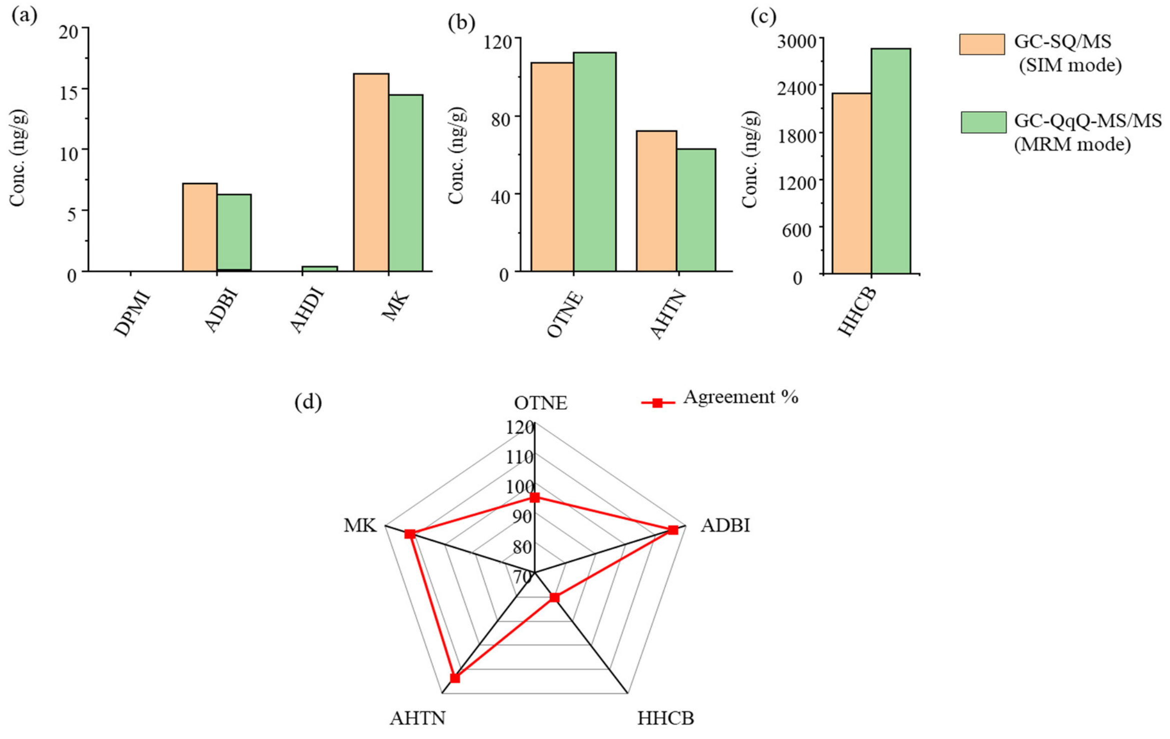

Figure S2. Comparison of individual SMCs quantifying results detected in a positive sample obtained by GC-SQ/MS with SIM mode and GC-QqQ-MS/MS with MRM mode. (a), (b) and (c) compare the results of detected SMCs according to the concentration range. (d) represents the percentage of agreement between two methods for the concentration results of the mainly detected SMCs.

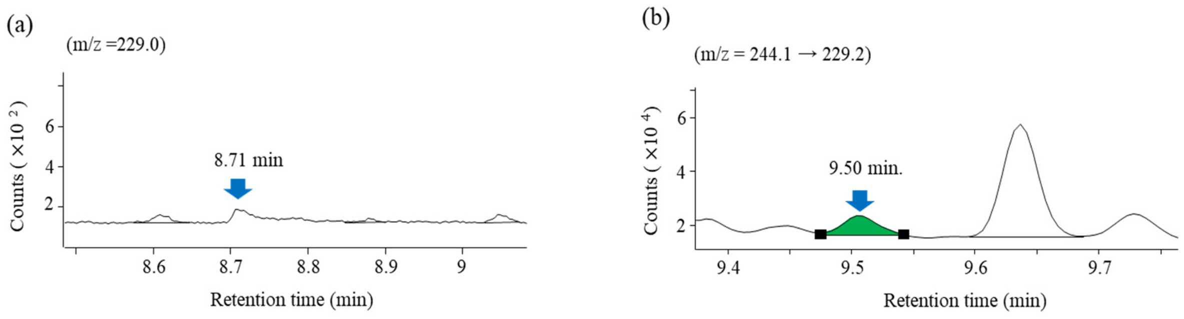

Figure S3. Chromatograms of AHDI obtained by (a) GC-SQ/MS with SIM mode (not detected < MDL) and (b) GC-QqQ-MS/MS with MRM mode at the concentration of 0.357 ng/g

(a) SIM mode (1 ng/μL)

(b) MRM mode (1 ng/μL)

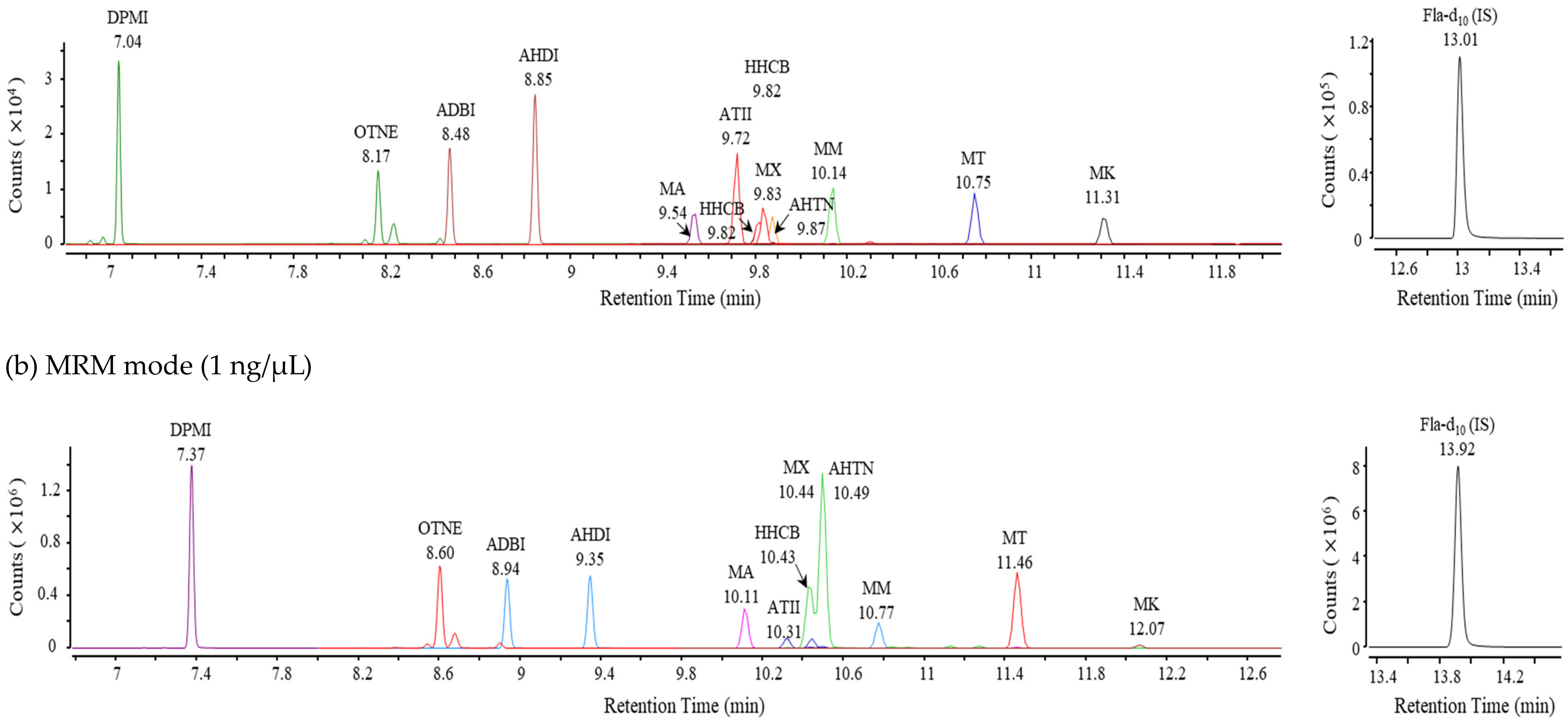

Figure S4. Typical chromatograms of the standard solutions with 12 SMCs and Fla-d_{10} (IS) obtained by GC-SQ/MS with SIM mode and GC-QqQ-MS/MS with MRM mode

Reference

1. Mottaleb, M.A.; Usenko, S.; O'Donnell, J.G.; Ramirez, A.J.; Brooks, B.W.; Chambliss, C.K. Gas chromatography–mass spectrometry screening methods for select UV filters, synthetic musks, alkylphenols, an antimicrobial agent, and an insect repellent in fish. *J. Chromatogr. A* **2009**, *1216*, 815-823.
2. Vallecillos, L.; Pocurull, E.; Borrull, F. Influence of pre-treatment process on matrix effect for the determination of musk fragrances in fish and mussel. *Talanta* **2015**, *134*, 690-698.
3. Castro, Ó.; Trabalón, L.; Schilling, B.; Borrull, F.; Pocurull, E. Solid phase microextraction Arrow for the determination of synthetic musk fragrances in fish samples. *J. Chromatogr. A* **2019**, *1591*, 55-61.
4. Trabalón, L.; Cano-Sancho, G.; Pocurull, E.; Nadal, M.; Domingo, J.L.; Borrull, F. Exposure of the population of Catalonia (Spain) to musk fragrances through seafood consumption: risk assessment. *Environ. Res.* **2015**, *143*, 116-122.
5. Subedi, B.; Mottaleb, M.A.; Chambliss, C.K.; Usenko, S. Simultaneous analysis of select pharmaceuticals and personal care products in fish tissue using pressurized liquid extraction combined with silica gel cleanup. *J. Chromatogr. A* **2011**, *1218*, 6278-6284.
6. Wang, T.; Zou, H.; Li, D.; Gao, J.; Bu, Q.; Wang, Z. Global distribution and ecological risk assessment of synthetic musks in the environment. *Environ. Pollut.* **2023**, *331*, 121893.
